# Tragus Nerve Stimulation Attenuates Postural Orthostatic Tachycardia Syndrome in Post COVID‐19 Infection

**DOI:** 10.1002/clc.70110

**Published:** 2025-02-27

**Authors:** Zhuo Wang, Tongjian Zhu, Xuping Li, Xin Lai, Mingxian Chen

**Affiliations:** ^1^ Department of Cardiology Renmin Hospital of Wuhan University Wuhan Hubei China; ^2^ Department of Cardiology, Institute of Cardiovascular Diseases Xiangyang Central Hospital, Afliated Hospital of Hubei University of Arts and Science Xiangyang Hubei China; ^3^ Department of Cardiology The Second Xiangya Hospital of Central South University Changsha Hunan China

**Keywords:** cardiovascular health, dysautonomia, Long COVID, neuropeptide Y, noninvasive neuromodulation

## Abstract

**Background:**

Postural orthostatic tachycardia syndrome (POTS) is characterized by symptoms of orthostatic intolerance and is frequently observed in post‐COVID conditions.

**Objectives:**

We conducted controlled, prospective, and randomized clinical trials to explore the potential therapeutic benefits of low‐level tragus stimulation (LL‐TS) in patients with POTS following COVID‐19 infection.

**Methods:**

This study enrolled 57 participants with confirmed post‐acute COVID‐19 who had been diagnosed with POTS. The ear clip was attached to the right tragus of the patients for stimulation (20 Hz with a 1‐ms duration) or sham stimulation. They were divided into two groups: the sham LL‐TS group (sham stimulation, *n* = 26) and the LL‐TS group (stimulation for 1 month, *n* = 31). LL‐TS was performed 1 h twice daily for 1 month. Postural tachycardia was evaluated at baseline, 1‐month visit, and 1‐year visit. Heart rate variability (HRV) and plasma neuropeptide Y (NPY) were evaluated at respective time points.

**Results:**

The mean age of participants was 31.9 ± 7.2 years (61.4% female). LL‐TS significantly attenuated the increase in heart rate from supine to a 10‐min stand, as well as the average and maximum heart rates after 1 month of treatment. LL‐TS also significantly reduced NPY levels. In addition, LL‐TS significantly increased the high frequency (HF), but decreased the low frequency (LF) and LF/HF ratio during the postural test (all *p* < 0.01). These effects persisted during the 1‐year follow‐up.

**Conclusion:**

LL‐TS may be a promising therapeutic approach for attenuating autonomic imbalance in patients with POTS following COVID‐19 infection.

## Graphical abstract

Schematic of the therapeutic effects of LL‐TS on POTS caused by SARS‐CoV‐2 via maintaining autonomic function. SARS‐CoV‐2 can cause autonomic dysfunction leading to POTS; however, LL‐TS has the potential to ameliorate POTS by enhancing vagal nerve activity and restoring autonomic function. LL‐TS = low‐level tragus stimulation; POTS = postural orthostatic tachycardia syndrome; SARS‐CoV‐2 = Severe Acute Respiratory Syndrome Coronavirus 2.

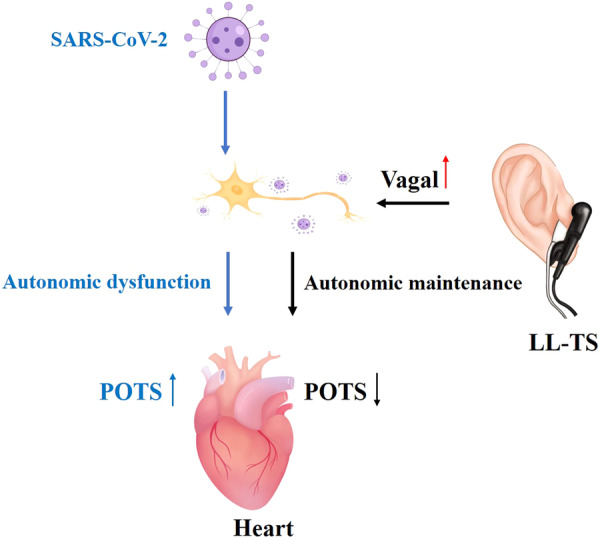

## 1 Introduction

The global impact of the COVID‐19 pandemic has extended beyond the acute phase, with a growing recognition of persistent symptoms and complications collectively termed as post‐acute sequelae of COVID‐19, or Long COVID [1]. Among the diverse symptoms observed in this prolonged phase, postural orthostatic tachycardia syndrome (POTS) has been frequently reported [2, 3, 4]. POTS is characterized by a sustained increase in heart rate upon standing, often accompanied by symptoms such as headache, cognitive impairment, nausea, and gastrointestinal dysmotility. Despite the availability of conventional pharmacological and nonpharmacological therapies, the current treatment options for POTS are limited by their efficacy and the prevalence of cardiac and noncardiac side effects [5, 6].

Patients with POTS may exhibit sympathetic over‐activity along with concurrent vagal impairment while in a supine position [7]. This profile leads to the hypothesis that any intervention resulting in an enhancement of parasympathetic activity and/or a decrease in sympathetic tone might attenuate POTS. Low‐level tragus stimulation (LL‐TS) is an innovative strategy for selectively activating afferent branches of the vagus nerve through electrical stimulation of specific areas of the external ear [8]. LL‐TS has emerged as a novel nonpharmacological approach for treating neuropsychiatric and cardiovascular disorders [9, 10]. Neuropeptide Y (NPY), being co‐released with norepinephrine from sympathetic nerve fibers, can indirectly reflect changes in sympathetic activity. LL‐TS targets the afferent branches of the vagus nerve to enhance parasympathetic activity while simultaneously reducing sympathetic overactivity. Assessing changes in NPY levels provides an indirect measure of the intervention's effect on sympathetic modulation. This study explores the potential effects of LL‐TS on the changes of heart rate (HR) and heart rate variability (HRV) in individuals with POTS following COVID‐19 infection.

### 1.1 Patients and Methods

This study included a total of 57 patients diagnosed with COVID‐19‐associated POTS from December 2019 to May 2023. This study was a multi‐center randomized study. The study protocol was approved by the Institutional Review Board of Wuhan Renmin Hospital of Wuhan University, Xiangyang Central Hospital, and the Second Xiangya Hospital of Central South University. All patients provided informed consent before enrollment in the study. The study complies with the Declaration of Helsinki, and the institutional Ethics Committee of Xiangyang Central Hospital reviewed and approved the study, waiving the requirement for patient consent (approval number: 2023‐018).

### 1.2 Inclusion Criteria

Patients were diagnosed with coronavirus disease 2019 (COVID‐19) based on positive results from SARS‐CoV‐2 polymerase chain reaction (PCR) testing. Patients eligible for enrollment in the study met the criteria for POTS after acute COVID‐19 infection, which was defined as an increase in HR of > 30 bpm within 10 min of standing from the supine position, without orthostatic hypotension (defined as a drop in blood pressure > 20/10 mmHg), exhibiting chronic symptoms lasting > 3 months and in the absence of any acute cause of orthostatic tachycardia [3, 4, 11]. All patients were randomly assigned to either the LL‐TS group or the sham LL‐TS group using a web‐based randomization table. In the LL‐TS group, tragus stimulation was performed using a device with an ear clip attached to the right‐sided tragus. In the sham LL‐TS group, sham stimulation was conducted using a similar device with an ear clip attached to the right‐sided tragus.

### 1.3 Exclusion Criteria

Patients were excluded from the study if they presented with any of the following conditions: significant hypertension, defined as systolic blood pressure > 150 mmHg or diastolic blood pressure > 100 mmHg in either supine or standing position, orthostatic hypotension characterized by a consistent drop in blood pressure > 20/10 mmHg within 10 min of standing, recent (< 6 months) history of stroke or myocardial infarction, significant immunological or hematological disorders, severe anemia, and history of vagotomy. Patients who received pharmacological treatment before undergoing LL‐TS were excluded from this study.

### 1.4 Low‐Level Tragus Stimulation

**Figure 1:**
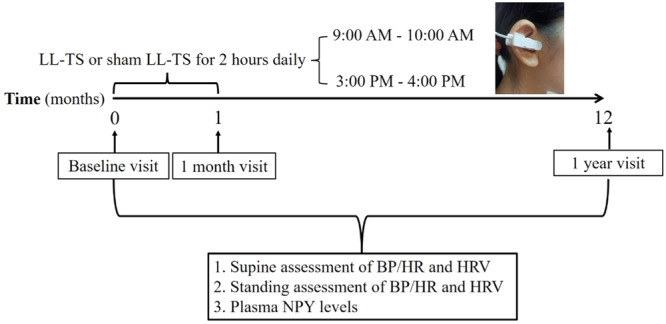
Schematic representation of the study design and timeline of events. A representative example of active stimulation, with the ear clip attached to the tragus is shown in the inset. BP indicates blood pressure; HR, heart rate; HRV, heart rate variability; LL‐TS, low‐level tragus stimulation; NPY, neuropeptide Y.

LL‐TS was administered to the tragus in the right ear according to previously established protocols [12]. An ear clip is attached to the right‐sided tragus, as shown in Figure 1. Incremental electric currents, set at 20 Hz with a 1‐ms duration, were delivered to the tragus using a stimulator (Rishena Co. Ltd, Changzhou City, Jiangsu Province, China) until a reduction in sinus rate was observed. The minimum electric current required to achieve this reduction was determined as the stimulation threshold. LL‐TS was then set at 50% below this threshold, with a duty cycle of 5 s on and 5 s off. Initiation of LL‐TS or sham LL‐TS commenced following the diagnosis of POTS in each patient. LL‐TS was performed twice daily, for 1 h each session, from 9:00 AM to 10:00 AM and from 3:00 PM to 4:00 PM, continuing for a total duration of 1 month. HR measurements were conducted at baseline and after 1 month (with or without LL‐TS). HR measurements were conducted following a 25‐min supine rest period and a subsequent 10‐min upright posture. These measurements were used to assess physiological responses to postural changes.

### 1.5 Heart Rate Variability Analysis

To calculate HRV, a 5‐min electrocardiogram using a DMS300‐4A system from DM Software Inc. was recorded in both the supine and upright positions. Patients were instructed to abstain from alcohol, smoking, and exercise for 12 h, and caffeine for 4 h before the visit to avoid any interference with the results. Data analysis was conducted using the HScribe Analysis System software from Mortara Instrument Inc., with a focus on heart rate and HRV frequency‐domain parameters. Specifically, HRV parameters, including LF and HF, were interpreted based on standard methods for HRV measurement. LF and HF components of HRV provide insights into sympathetic and parasympathetic autonomic activity, respectively, and their analysis is crucial for assessing autonomic function and its modulation following LL‐TS therapy. An electrocardiogram was recorded after the participant had remained in a supine position for 25 min and again after maintaining an upright posture for 10 min.

### 1.6 ELISA Measurement

A blood sample (20 mL) was collected after lying supine for 25 min, and then after standing for 10 min, respectively. Upon collection, samples underwent immediate processing by centrifugation at 3000 g for 15 min, followed by aliquoting and storage at −80°C until assay. NPY concentration was measured using a commercially available ELISA kit (ELK Biotechnology CO. Ltd, China), following the manufacturer's instructions.

### 1.7 Follow‐Up

Reassessment of patients’ HR, BP, symptom, plasma neuropeptide levels and HRV after 1 month of LL‐TS therapy at 1 year follow‐up.

### 1.8 Statistical Analysis

Continuous data were expressed as mean ± SD for variables with a normal distribution. Categorical data were presented as frequency and percentages, and comparisons between groups were conducted using Fisher's Exact method. For continuous data with a normal distribution, a two‐way ANOVA test was used for comparisons between groups. If the data did not follow a normal distribution, a non‐parametric alternative was used. All statistical analyses were performed using SPSS 22.0 software (SPSS Inc.). Statistical significance was set at *p* < 0.05.

## 2 Results

### 2.1 Baseline Patient Demographic and Clinical Data

Demographic and clinical characteristics of two groups are summarized in Supplementary Table 1. The Sham LL‐TS group consisted of 26 patients (9 males and 17 females) with a mean age of 31.0 ± 7.2 years. The LL‐TS group consisted of 31 patients (13 males and 18 females) with a mean age of 32.6 ± 7.4 years. As shown in Supplementary Table 1, there were no significant differences in age, BMI, smoking status, hypertension, hyperlipidemia, diabetes mellitus, asthma, or environmental allergy between the two groups. No side effects related to the device were observed.

### 2.2 The Effects of LL‐TS on the HR Increase and Peripheral NPY Levels in Patients With POTS

**Figure 2:**
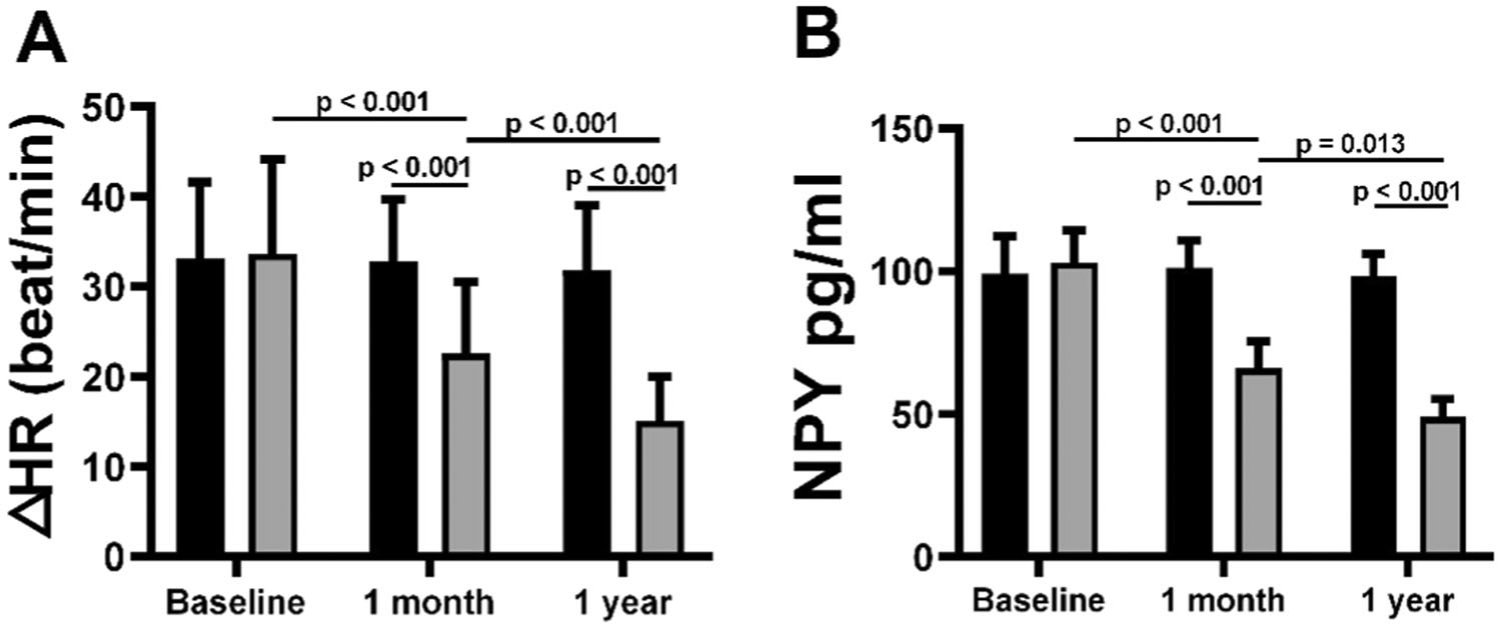
The effects of LL‐TS on the HR increase and peripheral NPY levels change in patients with POTS. (A) The increase in HR during the postural test was significantly attenuated at 1 month and 1 year in the LL‐TS group compared with the sham LL‐TS group (all *p* < 0.05). (B) LL‐TS significantly reduced the peripheral NPY levels at 1 month and 1 year during postural test compared with the sham LL‐TS group (all *p* < 0.05). ΔHR, HR at 10 min after the upright position ‐ HR after 25 min in the supine position; HF indicates high‐frequency; HR, heart rate; LF, low‐frequency; LL‐TS, low‐level tragus stimulation; NPY, neuropeptide Y; POTS, postural orthostatic tachycardia syndrome. *n* = 26 in the sham LL‐TS group, *n* = 31 in the LL‐TS group.

The increase in HR during the postural test was significantly attenuated at 1 month and 1 year in the LL‐TS group compared with the sham LL‐TS group (all *p* < 0.05), as shown in Figure 2. In the LL‐TS group, the increase in HR at 1 month was significantly reduced compared with that at the baseline (*p* < 0.05). In addition, the increase in HR at 1 year was significantly reduced compared with that at 1 month in the LL‐TS group (*p* < 0.05).

The peripheral NPY levels during the postural test were significantly reduced at 1 month and 1 year in the LL‐TS group compared with the sham LL‐TS group (all *p* < 0.05). In the LL‐TS group, the increase in HR at 1 month was significantly reduced compared with that at the baseline (*p* < 0.05). In addition, the increase in HR at 1 year was significantly reduced compared with that at 1 month in the LL‐TS group (*p* < 0.05).

### 2.3 The Effects of LL‐TS on the Blood Pressure During Postural Test in Patients With POTS

**Figure 3:**
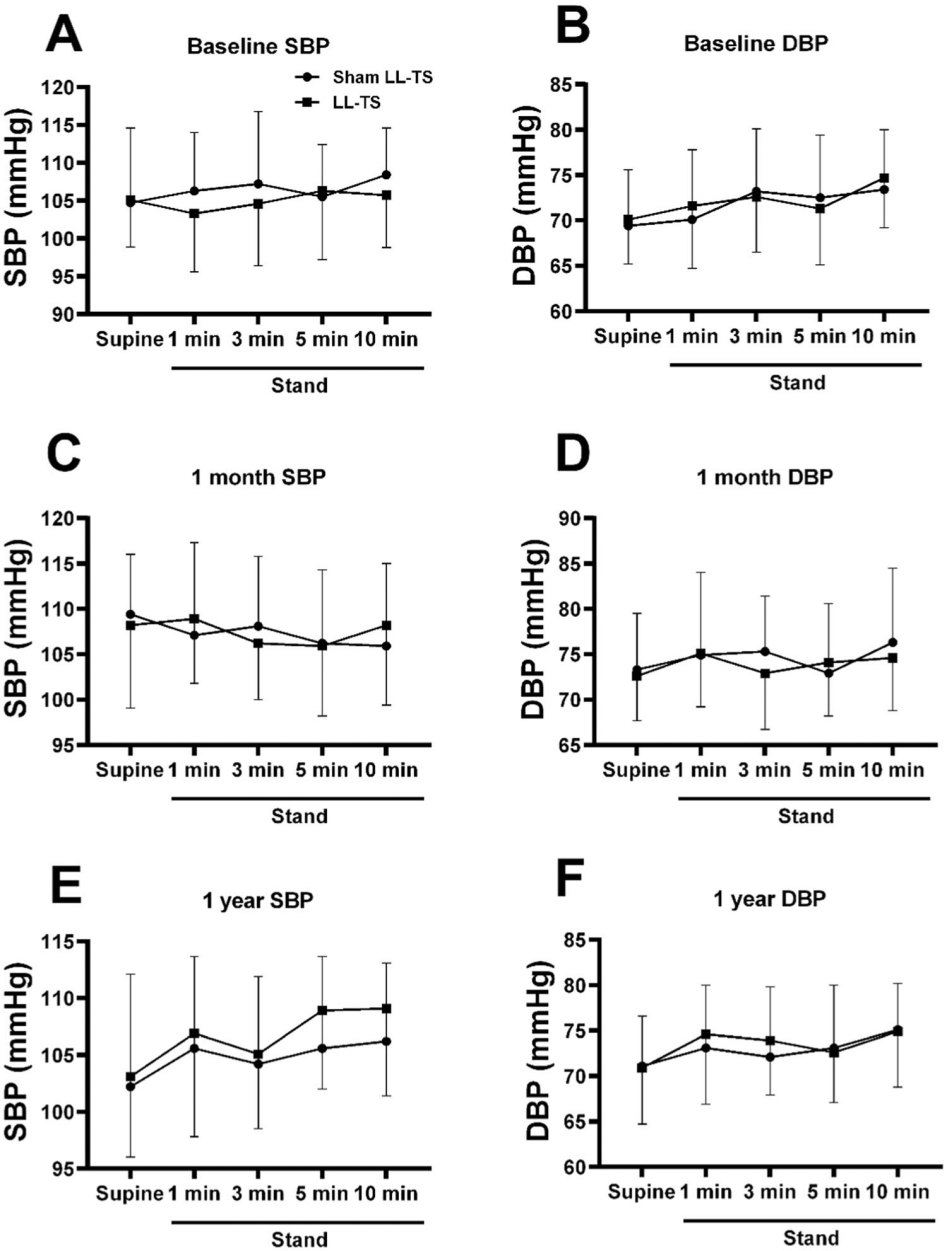
The effects of LL‐TS on the blood pressure in patients with POTS during postural test. No patient had a drop in both systolic blood pressure and diastolic blood pressure > 20 mmHg during the postural test at baseline (A and B), 1 month (C and D), or 1 year (E and F). DBP indicates diastolic blood pressure; LL‐TS, low‐level tragus stimulation; POTS, postural orthostatic tachycardia syndrome; SBP, systolic blood pressure. *n* = 26 in the sham LL‐TS group, *n* = 31 in the LL‐TS group.

No patient had a drop in both systolic blood pressure and diastolic blood pressure > 20 mmHg during the postural test, as shown in Figure 3.

### 2.4 The Effects of LL‐TS on HR Changes During the 24 h‐Holter

**Figure 4:**
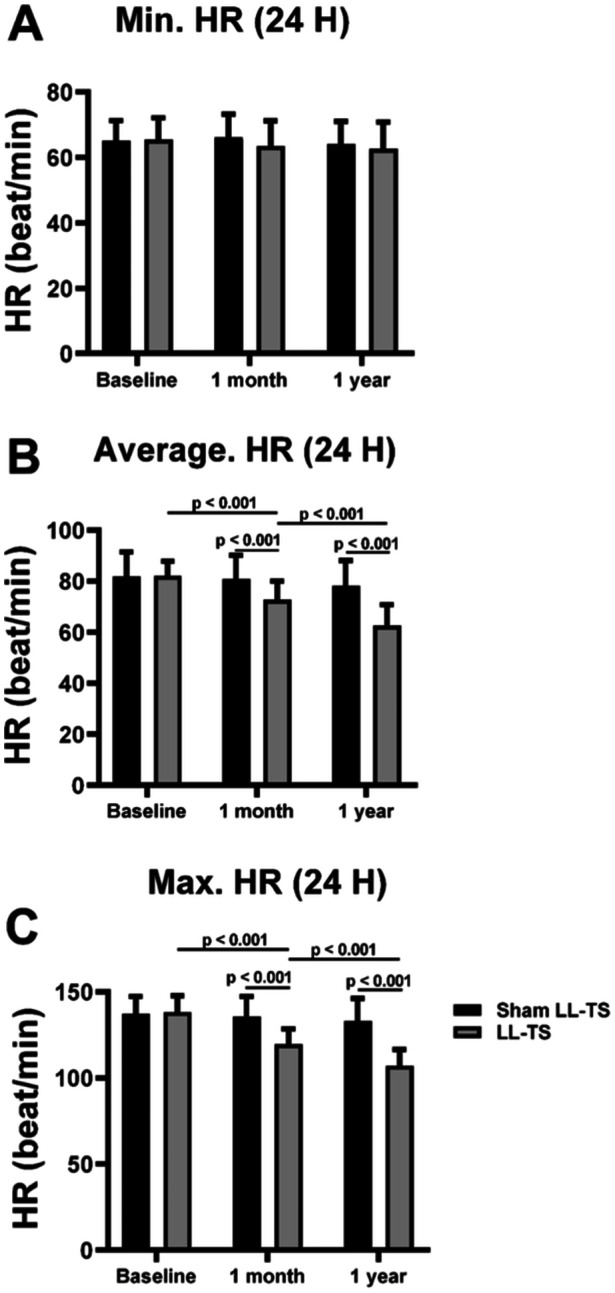
The effects of LL‐TS on HR during the 24 h‐holter. (A) LL‐TS had no effects on minimum HR during the 24 h‐holter. (B) LL‐TS significantly decreased the average HR during the 24 h‐holter. (C) LL‐TS significantly decreased the maximum HR during the 24 h‐holter. HR, heart rate; △HR, HR at 10‐min standing – HR at resting; LL‐TS indicates low‐level tragus stimulation; Max, maximum; Min, minimum. *n* = 26 in the sham LL‐TS group, *n* = 31 in the LL‐TS group.

There was no significant difference in minimum HR during the 24‐h Holter monitoring between the LL‐TS group and the sham LL‐TS group at baseline. The minimum and maximum HR were significantly decreased at 1 month and 1 year in the LL‐TS group compared with the sham LL‐TS group (all *p* < 0.05), as shown in Figure 4. In the LL‐TS group, the minimum and maximum HR at 1 month were significantly reduced compared with that at the baseline (*p* < 0.05). In addition, the minimum and maximum HR at 1 year were significantly reduced compared with that at 1 month in the LL‐TS group (*p* < 0.05).

### 2.5 The Effects of LL‐TS on HRV at Supine and Standing Condition

**Figure 5:**
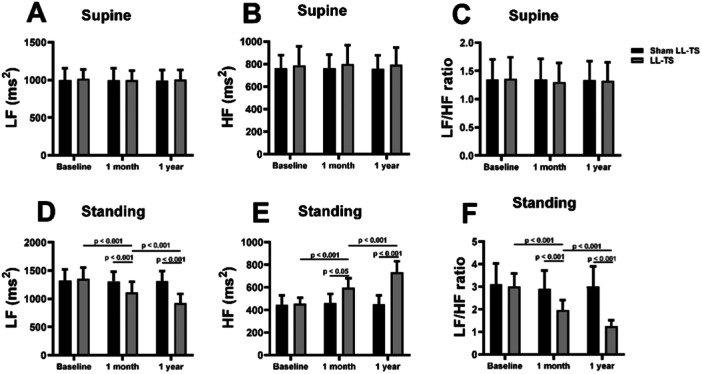
The effects of LL‐TS on HRV at supine and postural conditions. (A–C) LL‐TS had no effects on LF, HF, and LF/HF ratio at supine condition. (D–F) The change in LF and HF from supine to standing was significantly attenuated in the LL‐TS group compared with the sham LL‐TS group. Similarly, the postural change in the LF/HF ratio was significantly lower in the LL‐TS group compared with the sham LL‐TS group. HF indicates high‐frequency; LF, low‐frequency; LL‐TS, low‐level tragus stimulation. *n* = 26 in the sham LL‐TS group, *n* = 31 in the LL‐TS group.

HRV analysis was used to evaluate autonomic imbalances. There was no significant difference in LF and HF components between the sham LL‐TS group and the LL‐TS group during the supine position, as shown in Figure 5. However, the HF component of HRV, associated with parasympathetic activity, significantly increased from supine to standing in the LL‐TS group compared with the sham LL‐TS group (*p* < 0.001). Conversely, the LF component and the LF/HF ratio, which reflect sympathetic activity and the balance between sympathetic and parasympathetic activity, significantly decreased from supine to standing in the LL‐TS group compared with the sham LL‐TS group (*p* < 0.001).

## 3 Discussion

### 3.1 Major Findings

The major findings of this study are as follows. (1) LL‐TS reduces the heart rate response to the upright position in POTS patients. (2) POTS patients experience autonomic dysfunction, characterized by a decrease in parasympathetic activity and an increase in sympathetic activity after 10‐min standing. (3) LL‐TS could maintain the balance of autonomic activity and reduce NPY release. These findings suggest that LL‐TS may offer a therapeutic approach for reductions in upright heart rate response, alterations in autonomic imbalance, and decreases in NPY levels in individuals with POTS induced by post‐acute COVID‐19.

### 3.2 Autonomic Dysfunction and COVID‐19

While COVID‐19 primarily affects the respiratory system, it can also have systemic effects that extend beyond the acute phase of the illness. Some patients who have recovered from COVID‐19 may experience a range of persistent symptoms and complications, collectively known as post‐acute sequelae of COVID‐19 or Long COVID [13, 14, 15]. Among the diverse symptoms observed in this prolonged phase, POTS has been frequently reported. POTS is widely recognized as a complex disorder of the autonomic nervous system, characterized by orthostatic intolerance [16, 17]. Clinically, while POTS presents with orthostatic tachycardia as a hallmark feature, it also encompasses a broader range of symptoms associated with autonomic dysregulation, including headache, cognitive impairment, nausea, and gastrointestinal dysmotility. The potential mechanisms are likely related to an imbalance between sympathetic and parasympathetic activity, as well as hemodynamic abnormalities such as reduced systemic venous return, augmented splanchnic and extremity pooling, decreased cardiac output, and decreased cerebral perfusion. This dysregulation manifests as a decrease in parasympathetic tone and an increase in sympathetic activity, particularly during postural changes from supine to upright positions. HRV provides a noninvasive method to assess the interaction between the sympathetic and parasympathetic activity.

In HRV analysis, HF primarily represents vagal (parasympathetic) activity. High HF power indicates increased vagal tone and is associated with relaxation and restorative processes in the body. On the other hand, the low‐frequency (LF) component, along with the LF/HF ratio, is often interpreted as a marker of sympathetic activity. Higher LF power and LF/HF ratio are typically associated with increased sympathetic tone and arousal. In the present study, we observed a reduction in HF, and increases in LF and LF/HF ratio within 10 min of standing, accompanying postural changes from a recumbent to an upright position, highlighting the dysregulation of the ANS in patients with POTS. This dysregulation is typified by a decrease in parasympathetic activity and a concurrent increase in sympathetic activity, which are key contributors to the hallmark symptoms of POTS. Therefore, potentiation of parasympathetic response and inhibition of sympathetic drive may work as an effective therapeutic strategy in POTS.

### 3.3 LL‐TS and POTS

The tragus, a small protrusion in front of the ear canal, contains branches of the vagus nerve, a key component of the parasympathetic division of the ANS. LL‐TS involves the application of mild electrical stimulation to the tragus, which can selectively activate these afferent branches of the vagus nerve. LL‐TS is thought to enhance vagal (parasympathetic) tone by stimulating the afferent fibers of the vagus nerve. Increased vagal activity can lead to various physiological effects, including reduced heart rate and improved HRV [18]. While LL‐TS primarily targets vagal pathways, it may also exert indirect effects on sympathetic activity. By enhancing vagal tone, LL‐TS may lead to a relative decrease in sympathetic outflow, promoting autonomic balance [19, 20, 21, 22]. LL‐TS may also influence autonomic regulation through its effects on central nervous system structures involved in autonomic control. Electrical stimulation of the tragus has been shown to activate brain regions associated with autonomic function, including the nucleus tractus solitarius and the dorsal motor nucleus of the vagus [23]. Therefore, LL‐TS has emerged as a novel approach to modulating the ANS. In present study, we found the significant reduction in both average and maximum heart rate, as well as the attenuation of heart rate increase after a 10‐min standing period following LL‐TS intervention, suggests a favorable impact on heart rate regulation. Furthermore, the observed increase in HF, but decrease in LF and LF/HF ratio during standing conditions post‐LL‐TS intervention may indicate a rebalancing of sympathetic and parasympathetic activity, contributing to improved autonomic function. Therefore, that LL‐TS is a noninvasive neuromodulation in autonomic balance for individuals with POTS following post‐acute COVID‐19 infection.

### 3.4 NPY and POTS

NPY is a 36‐amino acid peptide neurotransmitter found in the brain and autonomic nervous system. It is involved in various physiological processes, including the regulation of energy balance, appetite, circadian rhythms, and stress responses [24]. NPY is co‐released with norepinephrine from sympathetic nerve endings. It enhances the release of norepinephrine, which binds to beta‐adrenergic receptors in the heart, leading to increased heart rate (positive chronotropic effect) [25]. NPY inhibits the release of acetylcholine from parasympathetic nerve endings. Reduced acetylcholine levels decrease the parasympathetic (vagal) tone of the heart, resulting in an increase in heart rate [26]. NPY potentially has effects on the changes of heart rate in patients with POTS. By modulating autonomic functions and brain activity, LL‐TS could potentially influence the pathways and systems where NPY is involved. In this study, we observed that LL‐TS significantly inhibited the plasma NPY levels. It indicated that LL‐TS could likely inhibit the release of NPY.

### 3.5 Limitations

While our study provides valuable insights into the potential therapeutic benefits of LL‐TS in patients with POTS following post‐acute COVID‐19 infection, several limitations should be acknowledged. First, the sample size in our study was relatively small, limiting the generalizability of our findings. Larger studies with a more diverse participant population are needed to confirm the efficacy and safety of LL‐TS in a broader range of patients with POTS. Second, this study primarily focused on the effects of LL‐TS on physiological parameters, such as reductions in upright heart rate response, alterations in HRV, and decreases in NPY levels, without directly assessing the broader clinical symptoms associated with POTS. Third, the study did not include a non‐POTS control group, such as a healthy cohort or individuals with long COVID but without POTS, which limits the ability to determine whether the observed effects of LL‐TS on upright heart rate response, HRV, and NPY levels are specific to POTS or generalizable to other conditions. Fourth, a limitation of this study is the lack of direct assessment of autonomic rebalancing, which has been proposed as a potential treatment for POTS. Future research should aim to evaluate the efficacy of such interventions to provide a more comprehensive understanding of their therapeutic potential.

## 4 Conclusions

LL‐TS represents a promising adjunctive therapy for individuals with POTS following post‐acute COVID‐19 infection, offering a noninvasive approach for reductions in upright heart rate response, alterations in HRV, and decreases in NPY levels.

## Conflicts of Interest

The authors declare no conflicts of interest.

## Supporting information

Supporting information.

## Data Availability

The data that support the findings of this study are available from the corresponding author upon reasonable request. The data are available from the corresponding author on reasonable request.
