# Supplementary material for: Tragus Nerve Stimulation Attenuates Postural Orthostatic Tachycardia Syndrome in Post COVID‐19 Infection
Source: Clin Cardiol. 2025 Feb 27;48(3):e70110. doi: 10.1002/clc.70110 (PMC11867165; doi:10.1002/clc.70110)
Supplement: Supplementary file 1 — Supporting information. [file CLC-48-e70110-s001.docx]

**Supplemental Table 1 Demographic and clinical characteristics**

|  | **Sham LL-TS group**  **(n = 26)** | **LL-TS group**  **(n = 31)** | **p value** |
| --- | --- | --- | --- |
| **Age (years)** | 31.0 ± 7.2 | 32.6 ± 7.4 | 0.36 |
| **Males, n (%)** | 9/26 (34.6%) | 13/31 (41.9%) | 0.60 |
| **Body mass index, mean±SD** | 23.8 ± 2.4 | 23.3 ± 2.2 | 0.52 |
| **Smoking, n (%)** | 3 (26.9%) | 5 (32.2%) | 0.72 |
| **Hypertension, n (%)** | 1 | 2 | 1.00 |
| **Hyperlipidemia, n (%)** | 7 | 11 | 0.57 |
| **Diabetes mellitus, n (%)** | 0 | 0 | 1.00 |
| **Asthma, n (%)** | 1 | 1 | 1.00 |
| **Environmental allergy, n (%)** | 1 | 2 | 1.00 |

Note: Data are presented as mean ± SD for continuous variables and as number (percentage) for categorical variables. LL-TS = Low-Level Tragus Stimulation; M = Male; BMI = Body Mass Index. P-values are derived from independent samples t-test for continuous variables and chi-square test for categorical variables.
